# Angiotensin-(1-7) Attenuates Kidney Injury Due to Obstructive Nephropathy in Rats

**DOI:** 10.1371/journal.pone.0142664

**Published:** 2015-11-10

**Authors:** Chang Seong Kim, In Jin Kim, Eun Hui Bae, Seong Kwon Ma, JongUn Lee, Soo Wan Kim

**Affiliations:** 1 Department of Internal Medicine, Chonnam National University Medical School, Gwangju, Korea; 2 Department of Physiology, Chonnam National University Medical School, Gwangju, Korea; University of Torino, ITALY

## Abstract

**Background:**

Angiotensin-(1–7) [Ang-(1–7)] counteracts many actions of the renin-angiotensin-aldosterone system. Despite its renoprotective effects, extensive controversy exists regarding the role of Ang-(1–7) in obstructive nephropathy, which is characterized by renal tubulointerstitial fibrosis and apoptosis.

**Methods:**

To examine the effects of Ang-(1–7) in unilateral ureteral obstruction (UUO), male Sprague-Dawley rats were divided into three groups: control, UUO, and Ang-(1–7)-treated UUO rats. Ang-(1–7) was continuously infused (24 μg/[kg·h]) using osmotic pumps. We also treated NRK-52E cells in vitro with Ang II (1 μM) in the presence or absence of Ang-(1–7) (1 μM), Mas receptor antagonist A779 (1 μM), and Mas receptor siRNA (50 nM) to examine the effects of Ang-(1–7) treatment on Ang II-stimulated renal injury via Mas receptor.

**Results:**

Angiotensin II (Ang II) and angiotensin type 1 receptor (AT_1_R) protein expression was higher in UUO kidneys than in controls. Ang-(1–7) treatment also decreased proapoptotic protein expression in UUO kidneys. Ang-(1–7) also significantly ameliorated TUNEL positive cells in UUO kidneys. Additionally, Ang-(1–7) reduced profibrotic protein expression and decreased the increased tumor growth factor (TGF)-β1/Smad signaling present in UUO kidneys. In NRK-52E cells, Ang II induced the expression of TGF-β1/Smad signaling effectors and proapoptotic and fibrotic proteins, as well as cell cycle arrest, which were attenuated by Ang-(1–7) pretreatment. However, treatment with A779 and Mas receptor siRNA enhanced Ang II-induced apoptosis and fibrosis. Moreover, Ang II increased tumor necrosis factor-α converting enzyme (TACE) and decreased angiotensin-converting enzyme 2 (ACE2) expression in NRK-52E cells, while pretreatment with Ang-(1–7) or A779 significantly inhibited or enhanced these effects, respectively.

**Conclusion:**

Ang-(1–7) prevents obstructive nephropathy by suppressing renal apoptosis and fibrosis, possibly by regulating TGF-β1/Smad signaling and cell cycle arrest via suppression of AT_1_R expression. In addition, Ang-(1–7) increased and decreased ACE2 and TACE expression, respectively, which could potentially mediate a positive feedback mechanism via the Mas receptor.

## Introduction

The renin-angiotensin-aldosterone system (RAAS) is an endocrine cascade that is a current target in the treatment of hypertension and prevention of cardiovascular or kidney disease. Angiotensin II (Ang II) is considered to be the main RAAS effector and exerts its activity via angiotensin II type 1 receptors (AT_1_R) and angiotensin II type 2 receptors (AT_2_R). In kidney disease, activation of the renal angiotensin-converting enzyme (ACE)/Ang II/AT_1_R axis results in deleterious effects, including vasoconstriction, hypertension, sodium and water retention, inflammation, fibrosis, and the generation of reactive oxidative species [1–3]. Therefore, current pharmacotherapeutic strategies have focused on inhibiting the production of Ang II (ACE inhibitors and direct renin inhibitors) or AT_1_R blockade [angiotensin receptor blockers (ARBs)] to delay the progression of renal diseases [4].

However, recent findings suggest that the ACE2/angiotensin-(1–7) [Ang-(1–7)]/Mas receptor axis may also play an important role in cardiovascular and kidney disease. Notably, ACE2/Ang-(1–7)/Mas receptor pathway activation exerts beneficial effects on cardiac remodeling and atherosclerosis, as well as experimental glomerulonephritis and diabetic nephropathy, which may be due to inhibition of fibrosis and reduced tumor growth factor (TGF)-β1 or reactive oxygen species accumulation [4–7]. In addition, genetic deletion of the Mas receptor may lead to fibrosis and renal dysfunction that are associated with AT_1_R and TGF-β1 upregulation [8]. Despite of the renoprotective effects of Ang-(1–7) in several kidney diseases, experiments using exogenously administered Ang-(1–7) in some preclinical models have yielded controversial results. For instance, Ang-(1–7) infusion in subtotal nephrectomy significantly increased blood pressure, cardiac hypertrophy, and fibrosis compared with vehicle-treated counterparts [9]. In addition, a recent study showed that Ang-(1–7) administration in mice with unilateral ureteral obstruction (UUO) exacerbates kidney injury when compared with controls, and also increased fibrosis, apoptosis, and inflammation in the obstructed kidneys of Mas receptor-deficient compared to wild-type counterparts [10]. Similarly, infusion of Ang-(1–7) in wild-type UUO mice caused more extensive renal injury than that seen in untreated mice. However, contrary to the previous study [10] the authors found that Mas receptor-deficient UUO mice were protected from this increase in apoptosis and inflammatory cell infiltration [11]. Therefore, the effects of the ACE2/Ang-(1–7)/Mas receptor cascade on obstructed kidney injuries are still conflicting and not entirely understood.

Thus, our study sought to determine the protective or deleterious effects of treatment of Ang-(1–7) in an obstructed kidney rat model. In addition, in vitro studies in NRK-52E rat tubular epithelial cells were designed to examine whether Ang-(1–7) treatment could alter the angiotensin receptor and ACE2 expression, in order to determine whether endogenous Ang-(1–7) is produced in response to Ang-(1–7) treatment. These results may further the knowledge to the pathophysiology of obstructive nephropathy and lead to novel treatment options for renal injury using Ang-(1–7).

## Materials and Methods

### Primary antibodies

The antibodies used were as follows: Anti-rabbit antibodies against TGF-β1 (3711), total Smad-2/3 (3102), phosphorylated Smad-2/3 (3101), Smad-4 (9515), Smad-6 (9519), Bax (2772), Bcl-2 (2870), total caspase-3 (9662), cleaved caspase-3 (9661), cyclin B1 (4138), phosphorylated cdc2 (Tyr 15) (9111), phosphorylated histone H3 (Ser 10) (3377; Cell Signaling Technology, Beverly, MA), AT_1_R (SC-1173; Santa Cruz Biotechnology, Santa Cruz, CA) and Mas receptor (AAR-013; Alomone Laboratories, Ltd., Jerusalem, Israel); anti-goat antibodies against connective tissue growth factor (CTGF) (SC-14939), ACE (SC-12187) and tumor necrosis factor-α converting enzyme (TACE) (SC-6416; Santa Cruz Biotechnology); anti-mouse antibodies against ACE2 (AF-3437; R&D systems, Minneapolis, MN), Ang II (NB100-62346; Novus Biochemicals, Littleton, CO), fibronectin (SC-71114; Santa Cruz Biotechnology, Santa Cruz, CA), α-smooth muscle actin (SMA) (A2547) and β-actin (A3854; Sigma) were commercially obtained.

### Animals

The experimental protocol was approved by the Institutional Animal Care and Use Committee of Chonnam National University Medical School. Studies were performed in male Sprague-Dawley rats weighing 180–200 g purchased from Samtako (Korea). Three days before UUO operation, rats were implanted with osmotic minipumps subcutaneously (*n* = 7, Alzet model 2002; Alza Corp., Cupertino, CA) for delivery of Ang-(1–7) (H-1715; Bachem Americas, Torrance, CA) at 24 μg/[kg·h] or vehicle (*n* = 7, sterile saline). The abdominal cavity was opened, and 2–0 silk ligature was placed at left proximal ureter under anesthesia with ketamine (50 mg/kg, i.p.). Control rats (*n* = 7) were treated in the same way, except that no ligature was made. The rats had free access to standard rat feed and tap water and were sacrificed 7 days after operation. They were considered to have a successful ureteral obstruction when ureteral dilatation was >2 mm and evidence of visible dilatation of renal pelvis in obstructed kidney compared with contralateral kidney was present [12]. The kidneys were quickly removed and the cortex/outer strip of the outer medulla (OSOM) isolated and stored at –70°C until use.

### Cell culture

NRK-52E cells were purchased from American Tissue Culture Collection (ATCC, Rockville, MD, USA). The cells were cultured with Dulbecco’s modified Eagle’s (DMEM; Sigma, St Louis, MO) containing 4.5 g/l glucose (Invitrogen, Carlsbad, CA), as previously described [13]. The cells were then treated using Ang II (1 μM, H-1705; Bachem Americas, Torrance, CA) with or without 1 h of pretreatment with Ang-(1–7) (1 μM) and Mas receptor antagonist A779 (1 μM, H-2888; Bachem Americas, Torrance, CA). The control cells were treated with a buffer solution alone. Mas receptor or Scramble small interfering RNA (siRNA) (4390711, 4390843; Ambion) was transfected in to NRK-52E cells using Lipofectamine 2000 reagent (Invitrogen) according to the manufacture’s instruction.

### Western blot analysis

The tissue was homogenized as previously described [14]. *In vitro*, the cells were harvested and washed twice with ice-cold PBS and re-suspended in lysis buffer and sonicated briefly. After centrifugation, the supernatant was prepared as protein extract. Equal amounts of protein were separated on 9 or 12% sodium dodecyl sulfate polyacrylamide gels. The blots were blocked with 5% milk in PBS-T for 1 h and then incubated overnight at 4°C with primary antibodies, followed by incubation with anti-rabbit or anti-mouse horseradish peroxidase-conjugated antibodies, as previously described [13]. All experiments were performed at least three times.

### Immunohistochemistry

Histological examination was performed as previously described [15]. The kidney was removed and cut into 2 mm-thick transverse sections, which were immersion fixed for 1 h, paraffin embedded, and incubated with the following primary antibodies: α-SMA, Bax, Bcl-2, TGF-β1 and AT_1_R.

### Terminal deoxynucleotidyl transferase-mediated dUTP nick-end labeling (TUNEL) assay

Apoptosis was assessed using the TUNEL assay and the numbers of apoptotic cells, as previously described [15]. Measurements utilized a ApopTag Plus Peroxidase *In Situ* Apoptosis Detection Kit (S7100; Millipore Corporation, Billerica, MA). TUNEL-positive cells were counted in the cortical tubular cells in ten x 100 fields per slide.

### Determination of reactive oxygen species generation

Intracellular reactive oxygen species (ROS) generation was measured with the fluoroprobe, 2′,7′-dichlorodihydrofluorescein diacetate (DCF-DA) (Molecular Probes, Wilmington, DE). At the end of the treatment period, the cells were incubated with 5 μM DCF-DA for 30 min at 37°C.

### Immunofluorescence

NRK-52E cells were cultured on chamber slide (Nalge Nunc International) and treated with Ang II (1 μM), Ang-(1–7) (1 μM) or A779, respectively. The primary antibody rabbit anti-AT_1_R was incubated with the cells overnight at 4°C in a humidified chamber. After washes, secondary antibody conjugated with Alexa Flour 568-labeled (red) goat anti-rabbit IgG (1:200 dilution; Invitrogen) was incubated with chamber slide for 1 h at room temperature. Stained cells were visualized using confocal laser microscope (LSM 510, Carl Zeiss, Germany), as previously described [13].

### Statistical analysis

Results are expressed as mean ± standard error of the mean (SEM). Statistical analyses were performed using the Mann-Whitney *U*-test or paired Student’s *t*-test, as appropriate. Data were considered statistically significant with values of *P*<0.05 (GraphPad Software, San Diego, CA).

## Results

### Effects of Ang-(1–7) on fibrosis and apoptosis in a UUO rat model

**Fig 1:**
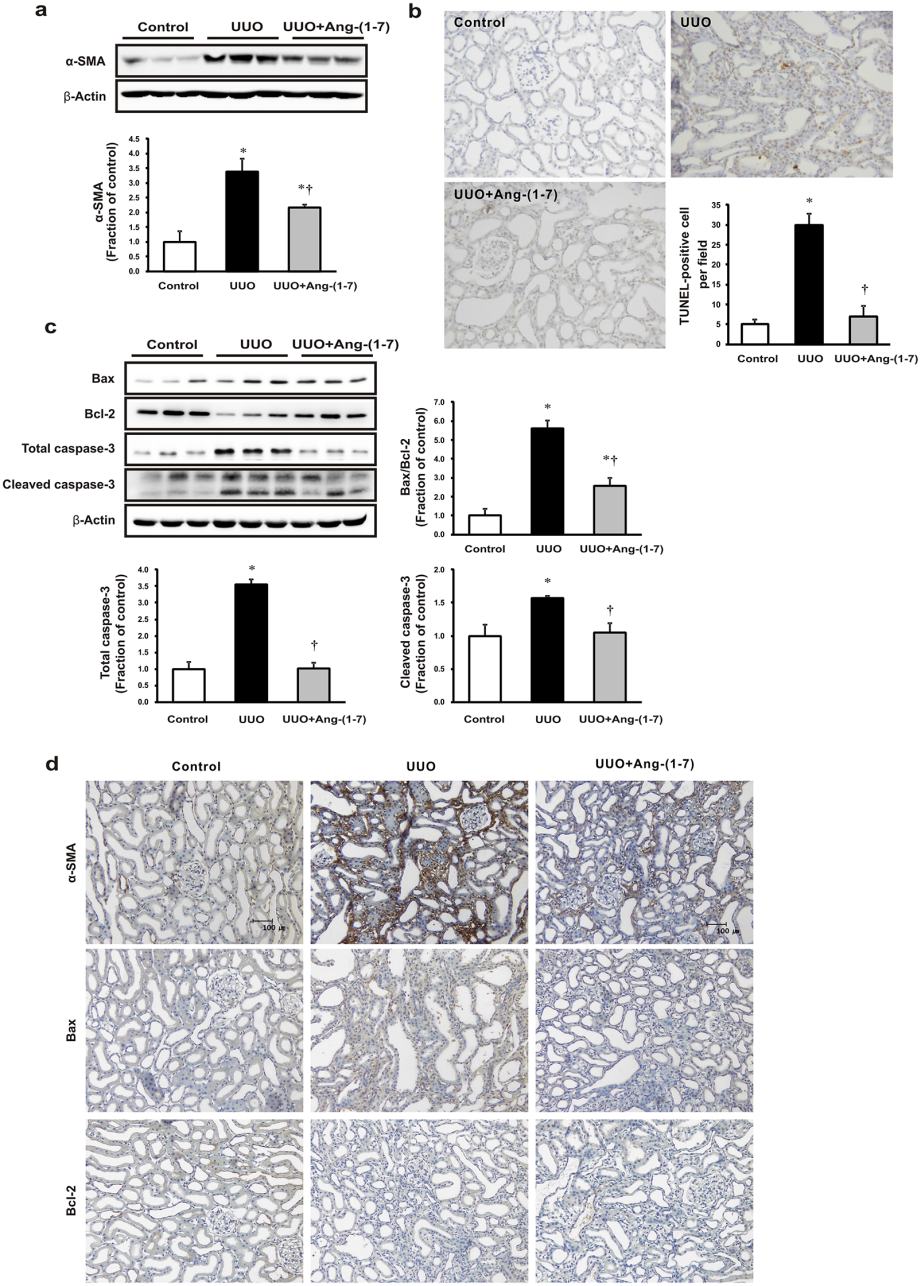
Effects of Ang-(1–7) on fibrosis and apoptosis in obstructed rat kidneys. Male Sprague-Dawley rats were subcutaneously implanted with osmotic minipumps to deliver Ang-(1–7) (24 μg/[kg·h]) or vehicle (sterile saline) 3 days before the unilateral ureteral obstruction (UUO) operation. The UUO lasted for 7 days. (a) α-smooth muscle actin (α-SMA) protein expression was assessed in UUO kidneys and unaffected controls. (b) Terminal deoxynucleotidyl transferase-mediated dUTP nick end-labeling (TUNEL) assays in kidneys from UUO rats following treatment with Ang-(1–7). (c) Prosurvival and proapoptotic protein expression was assessed in kidneys from UUO rats following treatment with Ang-(1–7). (d) Representative immunohistochemical staining of α-SMA, Bax, and Bcl-2 expression in UUO rats. Each column represents the mean ± SEM. Original magnification, 200×. *P < 0.05, compared with controls. †*P* < 0.05, compared with UUO rats.

We first investigated the effects of Ang-(1–7) on the expression of α-SMA, a molecular marker of myofibroblasts in obstructed kidneys. Fig 1a shows that α-SMA expression was higher in the obstructed kidneys than in the controls; the expression in obstructed kidneys decreased in response to treatment with Ang-(1–7) as compared to the levels in kidneys from vehicle-treated controls. We then performed a TUNEL assay to determine the effects of Ang-(1–7) on renal tubular apoptosis in UUO. Notably, we found an elevated number of tubular epithelial cells with TUNEL-positive nuclei in obstructed rat kidneys, and Ang-(1–7) treatment significantly attenuated cell apoptosis (Fig 1b). Moreover, western blot analysis revealed increased expression of the proapoptotic markers Bax and cleaved caspase-3, as well as attenuated expression of the antiapoptotic marker Bcl-2, resulting in an overall increase in the Bax/Bcl-2 ratio in obstructed kidneys compared with that in control rats. Similarly, these proapoptotic changes were restored by Ang-(1–7) treatment (Fig 1c). Immunohistochemistry analysis further confirmed the results obtained by western blotting (Fig 1d).

### Effects of Ang-(1–7) on TGF-β1/Smad signaling in UUO rat kidneys

**Fig 2:**
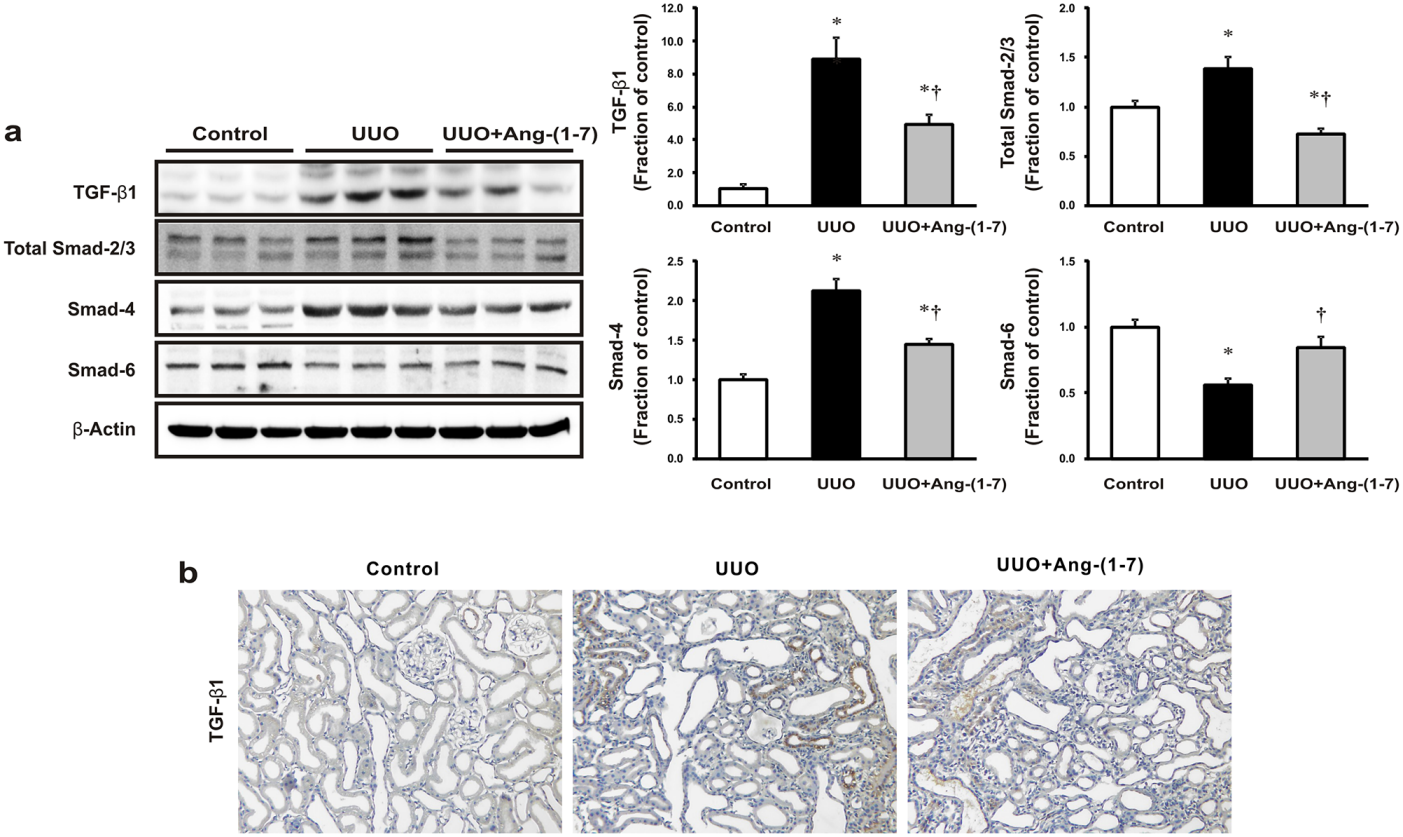
Effects of Ang-(1–7) on TGF-β1/Smad signaling in obstructed rat kidneys. (a) Tumor growth factor-β1 (TGF-β1) and Smad signaling was examined by western blot in UUO rat kidneys following treatment with Ang-(1–7). Each column represents the mean ± SEM. *P < 0.05, compared with controls. †*P* < 0.05, compared with UUO rats. (b) Representative immunohistochemical staining of TGF-β1 expression in UUO rats. Original magnification, 200×.

We next explored the expression of TGF-β1 and Smad signaling pathway effectors and their effect on renal tubular cell apoptosis and fibrosis. As shown in Fig 2a, the expression of TGF-β1, total Smad-2/3, and Smad-4 increased, whereas inhibitory Smad-6 expression was significantly lower in the obstructed kidneys than in controls. Again, these changes were significantly attenuated by treatment with Ang-(1–7). TGF-β1 immunostaining was increased in the obstructed kidneys as compared to controls, which was prevented by Ang-(1–7) treatment (Fig 2b).

### Alterations of ACE/Ang II/AT1R axis in UUO rats by Ang-(1–7)

**Fig 3:**
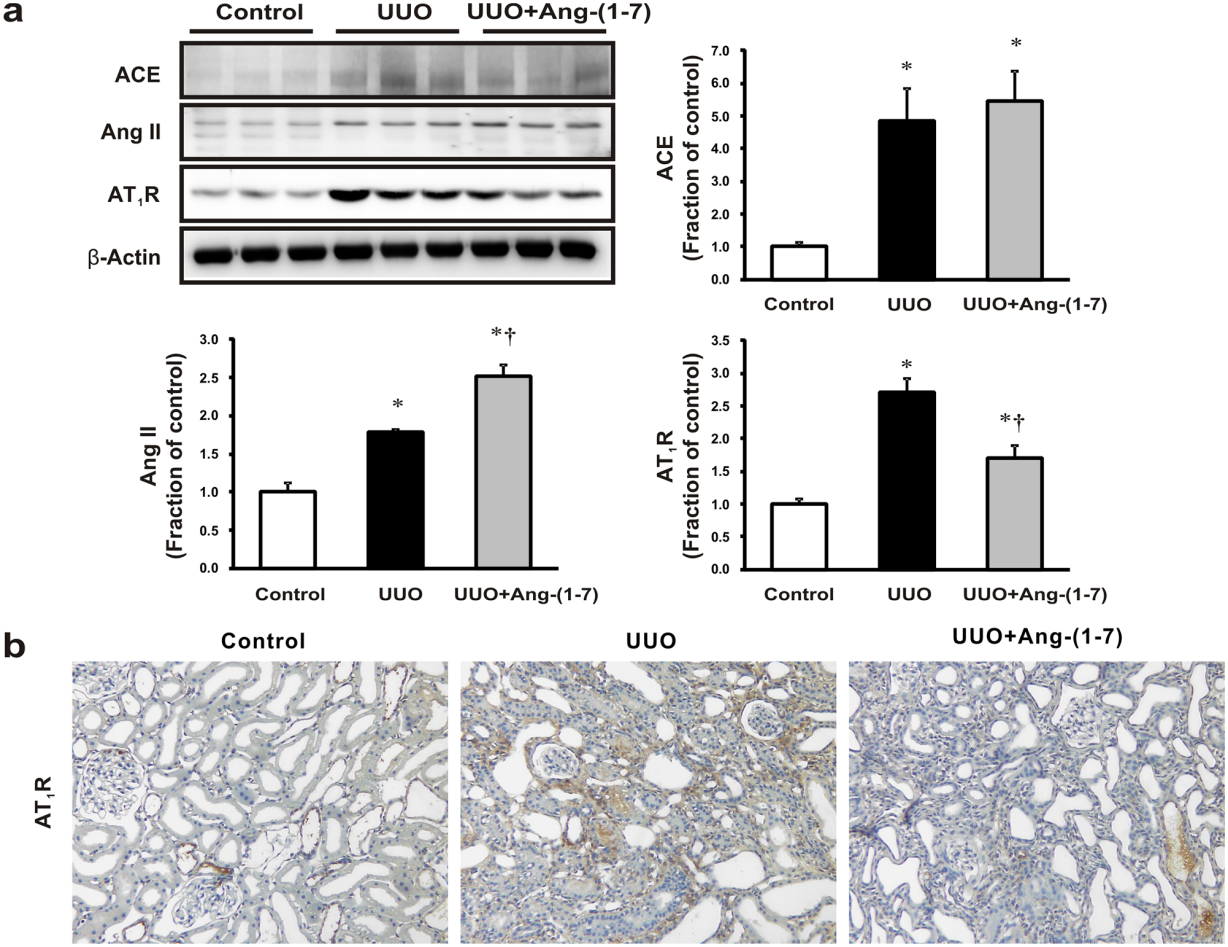
Effects of Ang-(1–7) on the ACE/Ang II/AT_1_R signaling axis in obstructed rat kidneys. (a) Angiotensin-converting enzyme (ACE), angiotensin II (Ang II), and angiotensin type 1 receptor (AT_1_R) protein expression was examined in UUO rat kidneys. Each column represents the mean ± SEM. *P < 0.05, compared with controls. †*P* < 0.05, compared with UUO rats. (b) Representative immunohistochemical staining of AT_1_R expression in UUO rats. Original magnification, 200×.

Next, we evaluated whether the ACE/Ang II/AT_1_R axis was altered in obstructed kidneys and also examined the subsequent effects of exogenous Ang-(1–7) treatment. Notably, the ACE/Ang II/AT_1_R axis was activated in obstructed rat kidneys when compared to control counterparts. Although no significant changes in ACE expression were observed in obstructed rat kidneys with or without exogenous Ang-(1–7), elevated Ang II expression was observed in obstructed rat kidneys in response to Ang-(1–7) treatment, which also significantly attenuated AT_1_R expression as compared to that in vehicle-treated obstructed rat kidneys by western blot and immunostaining (Fig 3a and 3b).

### Effects of A779 on angiotensin receptor expression, TGF-β1/Smad signaling and reactive oxygen species generation in Ang II-stimulated NRK-52E cells

**Fig 4:**
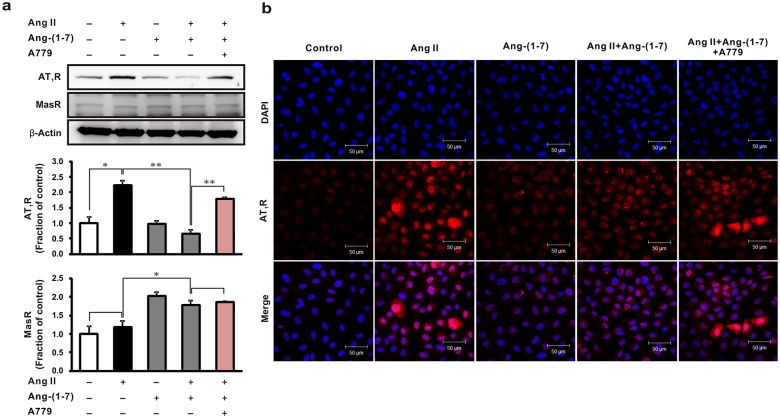
Effects of A779 on angiotensin receptors in Ang II stimulated rat tubular epithelial cells. (a) NRK-52E cells were exposed to Ang II (1 μM, 16 h) with or without 1 h pretreatment with Ang-(1–7) (1 μM) and Mas receptor antagonist A779 (1 μM). Each column represents mean ± SEM. *P < 0.05. **P < 0.01. Data are representative of at least three independent experiments. (b) AT_1_R expression (red) was examined in NRK-52E cells after treatment with Ang II, Ang-(1–7), and A779; nuclei were counterstained with 4,6-diamidino-2-phenylindole (DAPI) for immunofluorescence study. Original magnification, 400×. Scale bar = 50 μm. MasR, Mas receptor.

We subsequently performed in vitro studies to further explore the role of the Mas receptor in the cellular response to Ang-(1–7). Notably, AT_1_R expression was enhanced in Ang II-stimulated NRK-52E cells, and was blocked by pretreatment with Ang-(1–7) (Fig 4a). However, Ang-(1–7) did not block the Ang II-induced overexpression of AT_1_R in cells co-treated with A779, suggesting that Ang II-stimulated AT_1_R expression was attenuated by Ang-(1–7) in a Mas receptor-dependent manner. Immunofluorescence analysis confirmed that A779 restored the expression of AT_1_R, which was subsequently inhibited by treatment with exogenous Ang-(1–7) in Ang II-stimulated cells (Fig 4b). Moreover, we also determined that Mas receptor expression increased after pre-treatment with Ang-(1–7) in cells, but was not altered by that of Ang II or A779.

**Fig 5:**
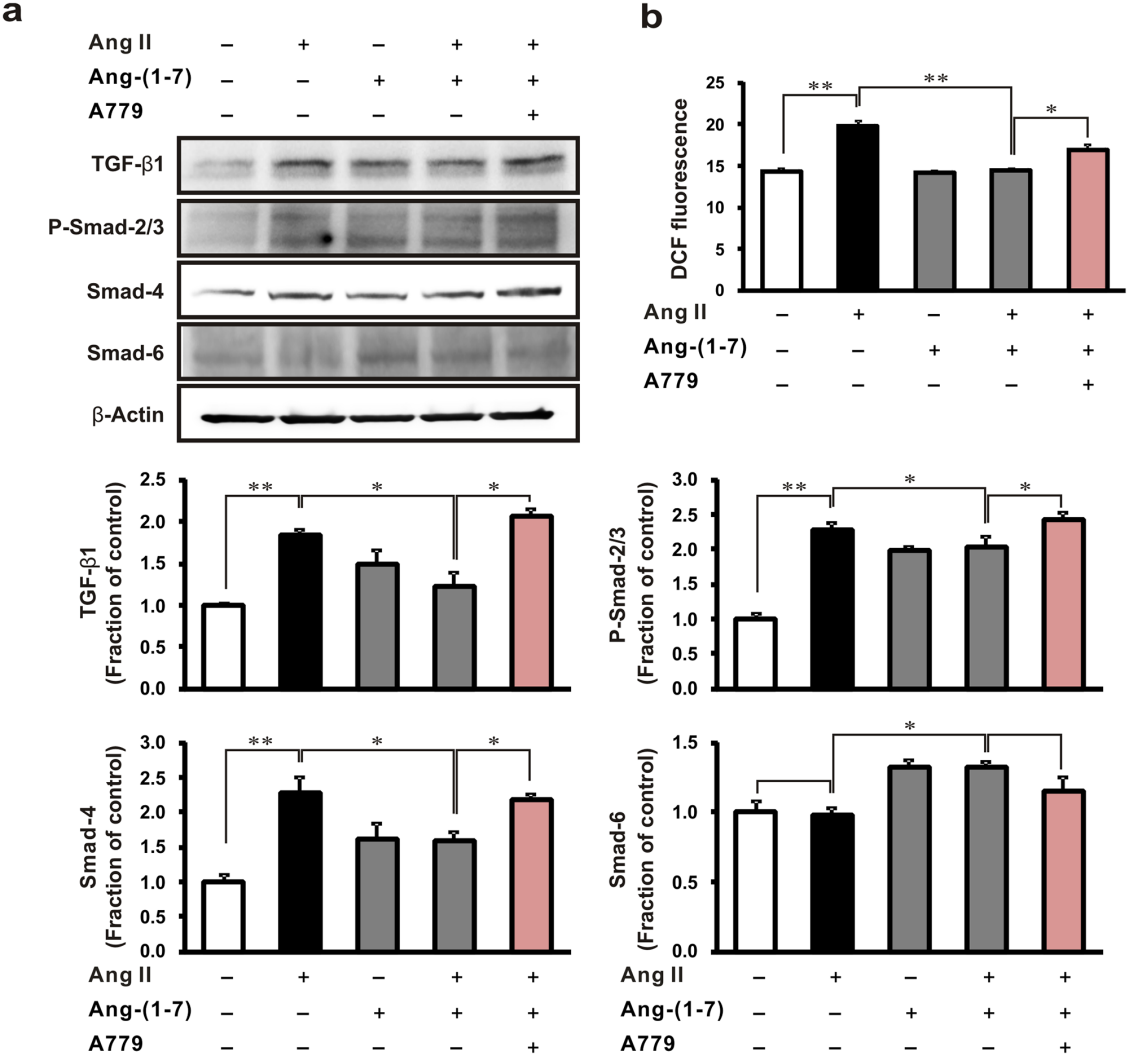
Effects of A779 on TGF-β1/Smad signaling and *ROS generation* in Ang II-stimulated rat tubular epithelial cells. NRK-52E cells were exposed to Ang II (1 μM, 16 h) with or without 1 h pretreatment with Ang-(1–7) (1 μM) and Mas receptor antagonist A779 (1 μM). (a) TGF-β1/Smad expression was analyzed. (b) The generation of reactive oxygen species (ROS) was detected using the ROS-sensitive fluorescent dye, DCF-DA. Each column represents the mean ± SEM. *P < 0.05. **P < 0.01. Data are representative of at least three independent experiments.

We also analyzed TGF-β1-medicated Smad signaling in NRK-52 cells following treatment with or without Ang-(1–7) or A779. Notably, incubation with Ang II increased the expression of TGF-β1, p-Smad-2/3, and Smad-4 in cells, all of which were attenuated by pretreatment with Ang-(1–7). Co-treatment with A779 abolished the effect of Ang-(1–7), corresponding to previous results (Fig 5a).

In addition, ROS generation was detected using the ROS-sensitive fluorescent dye DCF-DA. Notably, the Ang II-induced increase in DCF-DA fluorescence intensity was attenuated by Ang-(1–7), while co-treatment with A779 abolished these effects (Fig 5b).

### Effects of A779 on fibrosis and apoptosis in Ang II-stimulated NRK-52E cells

**Fig 6:**
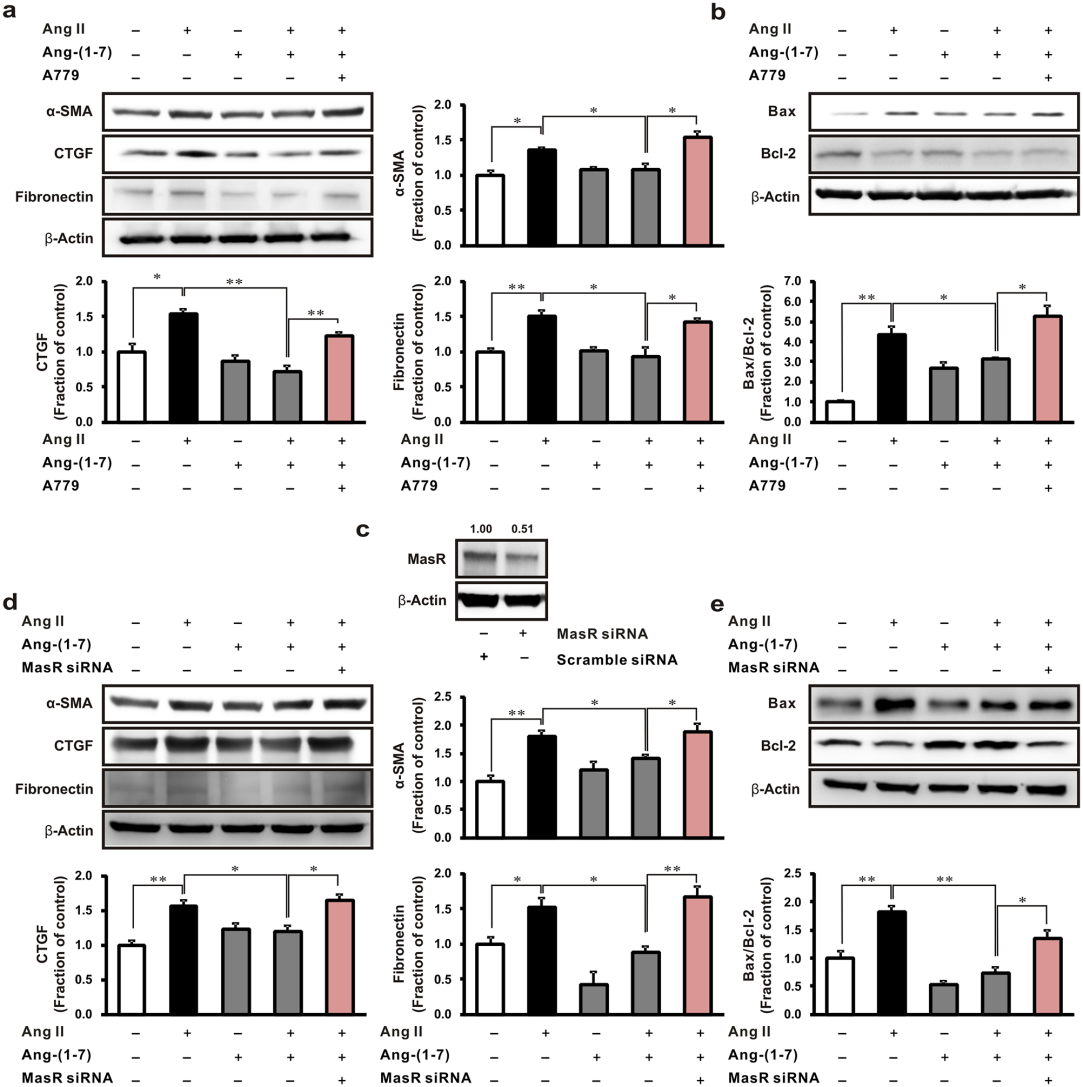
Effects of A779 on fibrosis and apoptosis in Ang II-stimulated rat tubular epithelial cells. NRK-52E cells were exposed to Ang II (1 μM, 16 h) with or without 1 h pretreatment with Ang-(1–7) (1 μM) and Mas receptor antagonist A779 (1 μM). (a) α-smooth muscle actin (α-SMA), connective tissue growth factor (CTGF), and fibronectin, as well as (b) the Bax and Bcl-2 protein expressions were then analyzed. (c) Downregulation of Mas receptor protein in NRK-52E cells at 48 h after Mas receptor siRNA transfection. NRK-52E cells were transfected with Mas receptor siRNA and then exposed to Ang II (1 μM, 16 h) with or without 1 h pretreatment with Ang-(1–7) (1 μM). Western blot analyses represented the (d) α-SMA, CTGF and fibronectin, as well as (e) the Bax and Bcl-2 protein expression. Each column represents the mean ± SEM. *P < 0.05. **P < 0.01. Data are representative of at least three independent experiments.

We assessed the role of the Mas receptor in fibrosis and apoptosis in Ang II-stimulated NRK-52E cells. Pretreatment with Ang-(1–7) significantly inhibited Ang II-induced α-SMA, CTGF, and fibronectin expression, while the effects of Ang-(1–7) were reversed by the treatment with A779 (Fig 6a). In addition, Ang II increased Bax expression while inhibiting that of Bcl-2, resulting in an increased Bax/Bcl-2 ratio in NRK-52E cells. As expected, this ratio was lowered in response to by pretreatment with Ang-(1–7) in Ang II-incubated cells, and was reversed by the addition of A779 (Fig 6b). As shown in Fig 6c, Mas receptor protein expression was downregulated about 49% in cells transfected with Mas receptor siRNA (50 nM) compared to those with scramble siRNA at 48 h after transfection. Ang-(1–7) decreased the expression of Ang II-stimulated profibrotic and proapoptotic protein, while Mas receptor siRNA transfection could markedly increase their expression (Fig 6d).

### Effects of A779 on cell cycle arrest in Ang II-stimulated NRK-52E cells

**Fig 7:**
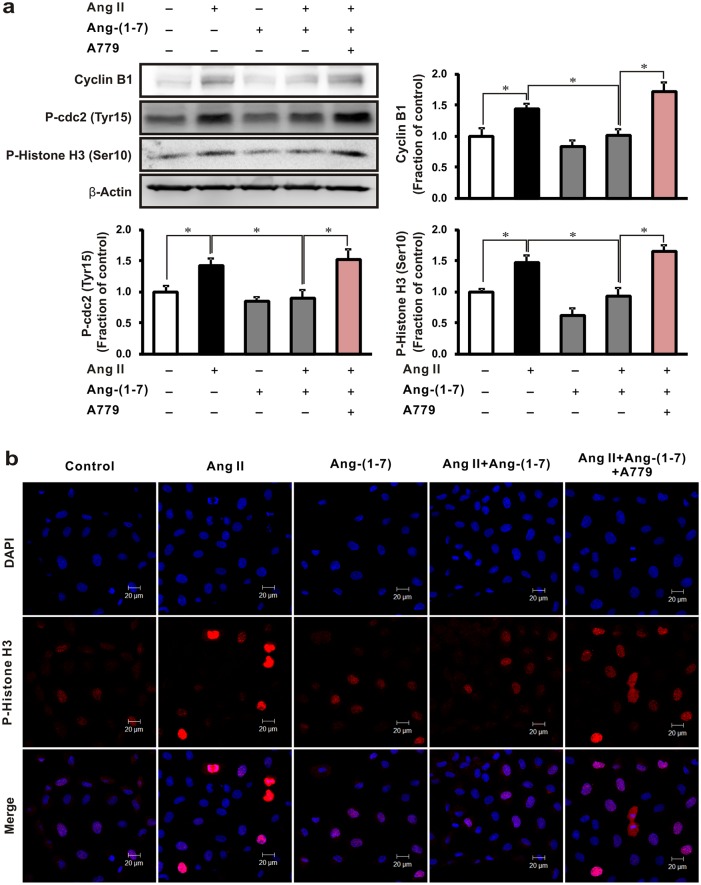
Effects of A779 on cell cycle arrest in Ang II-stimulated rat tubular epithelial cells. (a) Ang II (1 μM, 16 h) with or without 1 h pretreatment with Ang-(1–7) (1 μM) and Mas receptor antagonist A779 (1 μM). Each column represents the mean ± SEM. *P < 0.05. Data are representative of at least three independent experiments. (b) Phosphorylated histone H3 (Ser 10) expression (red) was examined in NRK-52E cells after treatment with Ang II, Ang-(1–7), and A779; nuclei were counterstained with 4,6-diamidino-2-phenylindole (DAPI) for immunofluorescence study. Original magnification, 400×. Scale bar = 20 μm.

We then investigated whether Ang-(1–7) influenced cell cycle arrest in Ang II-stimulated NRK-52E cells. Expression of the G2/M progression markers cyclin B1, phosphorylated Cdc-2 (Tyr 15), and histone H3 (Ser 10) increased after treatment of Ang II, and were reduced by pretreatment of Ang-(1–7). However, the effects of Ang-(1–7) were reversed by the treatment with A779 (Fig 7a). We also confirmed that the similar effects of Ang-(1–7) were reversed by A779 treatment in immunofluorescence analysis (Fig 7b).

### Effects of exogenous Ang-(1–7) on ACE2 and TACE expression in Ang II-stimulated NRK-52E cells

**Fig 8:**
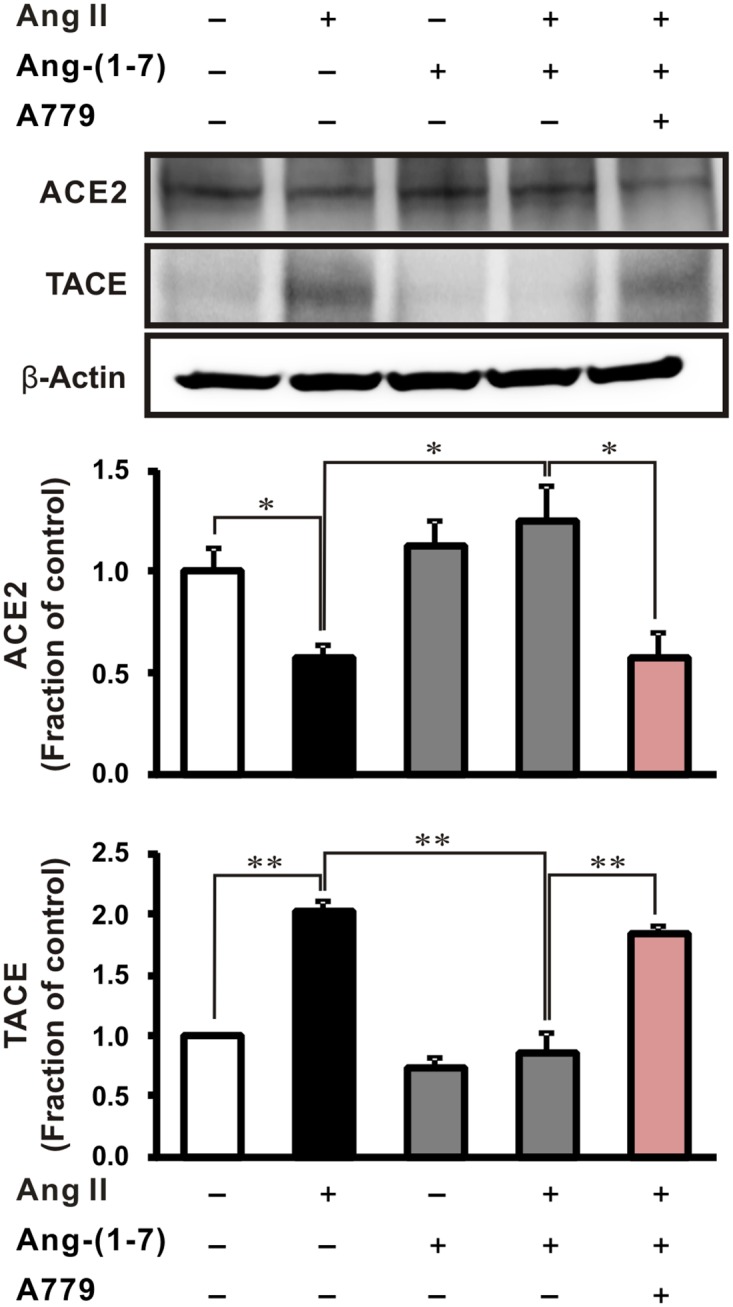
Effects of exogenous Ang-(1–7) on ACE2 and TACE expression in Ang II-stimulated rat tubular epithelial cells. NRK-52E cells were exposed to Ang II (1 μM, 16 h) with or without 1 h pretreatment with Ang-(1–7) (1 μM) and Mas receptor antagonist A779 (1 μM). Each column represents the mean ± SEM. *P < 0.05. **P < 0.01. Data are representative of at least three independent experiments.

A recent study showed that Ang II suppresses ACE2 expression by increasing TACE activity and ACE2 cleavage in the heart in an AT_1_R-dependent manner [16]. Hence, elevated TACE activity likely represents a loss of the protective effects of endogenous Ang-(1–7) that is generated by ACE2. Therefore, to determine whether exogenous Ang-(1–7) affects endogenous Ang-(1–7) activity, we evaluated the expression of ACE2 and TACE in Ang II-treated NRK-52E cells. As shown Fig 8, Ang II increased the expression of TACE and decreased ACE2 in cells, while pretreatment with Ang-(1–7) significantly inhibited these effects in Ang II-treated cells. Accordingly, pretreatment with A779 significantly increased the expression of TACE and decreased that of ACE2 by blocking the effects of Ang-(1–7). Altogether, these results indicated that exogenous Ang-(1–7) could potentially mediate and increase of endogenous Ang-(1–7) via Mas receptor activity.

## Discussion

The present study demonstrated that Ang-(1–7) treatment significantly improves kidney injury by mediating antifibrosis and antiapoptosis processes in an obstructed rat kidney model. Our main finding is that enhanced ACE/Ang II/AT_1_R pathway activity in the obstructed kidney promotes TGF-β1/Smad-dependent signaling, resulting in the increased expression of fibrotic and apoptotic markers. However, Ang-(1–7) attenuated these renal injures via suppression of AT_1_R expression and ROS generation and recovery of G2/M cell cycle arrest in a Mas receptor-dependent manner. Furthermore, Ang-(1–7) increased endogenous ACE2 expression that results from AT_1_R-mediated TACE suppression, which suggested that treatment with exogenous Ang-(1–7) may also induce the increase of endogenous Ang-(1–7) in renal tubular cells.

Among the non-classical RAAS, the ACE2/Ang-(1–7)/Mas receptor axis generally opposes the actions of the ACE/Ang II/AT_1_R axis through nitric oxide-dependent effects, including mediates vasodilatation, natriuresis, dieresis, and diminished oxidative stress [17, 18]. Indeed, the beneficial effects of Ang-(1–7) with regard to cardiovascular or kidney diseases have been widely demonstrated in animal studies [19–25]. Consistent with our findings, previous studies demonstrate that treatment with Ang-(1–7) protects the kidney by limiting glomerulosclerosis, proteinuria, inflammation, fibrosis, and apoptosis in various experimental models, such as those of chronic kidney disease, diabetes, and nephrotic syndrome [23–25].

On the other hand, several controversial findings concerning the pathophysiological role of Ang-(1–7) still exist in similar kidney diseases models [9, 25–27]. For instance, Esteban *et al*. showed that Mas receptor-deficient mice with renal disease exhibit attenuated renal damage as determined by lower overall fibrosis, apoptosis, and inflammatory cell infiltration in obstructed or ischemia reperfusion injury kidney when compared to wild-type mice [11]. Moreover, Ang-(1–7)-treated wild-type mice display increased the apoptosis, leukocyte infiltration, and matrix deposition in the mesangial area as compared to that in untreated counterparts [11]. In a recent study, Zimmerman *et al*. reported that Ang-(1–7) infusions exacerbate kidney injury by promoting fibronectin, α-SMA, and TGF-β1 expression, as well as NADPH oxidase activity, in obstructed mouse kidneys, whereas those from Mas receptor-deficient mice exhibited enhanced apoptosis and macrophage infiltration than that of with wild-type mice. Altogether, these data suggest that not exogenous but endogenous Ang-(1–7) exerts renoprotective effects via interactions with the Mas receptor [10]. These discrepancies regarding the role of Ang-(1–7) might be related to stimulation of other angiotensin receptors (non-Mas receptors) and differences in Ang-(1–7) dose, duration, and administration route, or the preclinical animal species [4, 28].

Although several studies demonstrate conflicting roles on exogenous Ang-(1–7) treatment in renal disease progression, previous UUO mouse studies [10, 11] did not detail any effects resulting from alterations in AT_1_R expression. AT_1_R is considered to be a therapeutic target in the clinical setting, and receptor-receptor interactions between AT_1_R and Mas receptor are thought to be important in renal disease. In the present study, AT_1_R expression was higher in obstructed kidneys than in unobstructed kidneys and was inhibited by treatment with Ang-(1–7). However, no significant changes in ACE expression were observed with or without Ang-(1–7) treatment, whereas Ang II expression increased after treatment with Ang-(1–7) in our model. Therefore, the downregulation of AT_1_R might play an important role in the renoprotective effects, rather than the level of ACE and Ang II [29].

In the context of the mechanism by which Ang-(1–7) protects against kidney injury due to obstruction, AT_1_R expression could be regulated via the Mas receptor. Pinheiro *et al*. previously reported that AT_1_R mRNA expression was higher in Mas receptor-knockout mice than in wild-type controls [8]. Meanwhile, the formation of receptor complexes has gained significant attention in recent years [30]. Indeed, the Mas receptor acts as an AT_1_R antagonist through the constitutive formation of hetero-oligomeric complexes in transfected mammalian cell lines [31]. Consistent with these observations, our in vitro experiments demonstrated that the upregulation of AT_1_R induced by Ang II treatment could be attenuated by pretreatment with Ang-(1–7). However, co-treatment with Mas receptor antagonist A779 increased the expression of AT_1_R. Our immunofluorescence experiments also showed that AT_1_R expression increased following A779 treatment. Taken together, our data indicated that the ACE/Ang II/AT_1_R-induced kidney damage observed in the obstructed rat kidney could be prevented by hindering AT_1_R expression with Ang-(1–7) treatment in a Mas receptor-dependent manner.

Ang II and TGF-β1 are considered to two major factors that mediate fibrogenesis in the kidney [32–34]. Ang II increases during obstructive nephropathy and promotes renal fibrosis by directly stimulating TGF-β1 expression or via AT_1_R activation [35, 36]. In addition, Ang II-induced apoptosis is mediated via AT_1_R and AT_2_R though TGF-β1, followed by the expression of Fas, Fas ligand, Bax and the activation of caspase-3 [37]. Most of all, TGF-β1 is a potent renal profibrotic and proapoptic factor associated with RAAS activation in kidney disease. Moreover, proximal tubular cells arrested in the G2/M phase of the cell cycle promoted fibrosis with enhanced TGF-β1 and CTGF gene transcription in an obstructive model of acute kidney injury [38]. Cell cycle regulation at the G1/S or G2/M phases has been associated with programed cell death [39]. In line with previous studies, our results showed that treatment with Ang-(1–7) modulated the TGF-β/Smad signaling and cell cycle arrest, which involved in fibrosis and apoptosis, in obstructive kidneys.

Over the last decade, ACE2 has emerged as a key player in the pathophysiology of hypertension and cardiovascular and renal disease due to its pivotal role in metabolizing Ang II into more favorable Ang-(1–7). Therefore, the ACE2 expression or activity is an important issue in regards to the generation of endogenous Ang-(1–7). A recent study reported that endogenous Ang-(1–7) exerts its protective effects against kidney fibrosis, apoptosis, oxidative stress, and inflammation in UUO mice via interactions with the Mas receptor [10]. Interestingly, our in vitro data indicated that exogenous Ang-(1–7) increased ACE2 expression, which might also be associated with Mas receptor-mediated TACE suppression. These results could be explained by the downregulation of AT_1_R in response to treatment with Ang-(1–7), which leads to the inactivation of TACE and the subsequent breakdown of ACE2 [16]. Taken together, while endogenous Ang-(1–7) was not measured in our study, this evidence suggests that exogenous Ang-(1–7) may induce an increase in endogenous Ang-(1–7). Consequently, both exogenous and endogenous Ang-(1–7) likely exert synergic effects to prevent kidney damage in a Mas receptor-dependent manner.

## Conclusions

The results presented in this study demonstrated that infusion with Ang-(1–7) prevents obstructive nephropathy by suppressing renal apoptosis and fibrosis, possibly through the inhibition of TGF-β1/Smad signaling and recovery of G2/M cell cycle arrest, and the subsequent suppression of AT_1_R expression. Moreover, exogenous Ang-(1–7) increased ACE2 expression, which could potentially mediate an increase in endogenous Ang-(1–7) in a positive feedback mechanism via the Mas receptor. Although the role of exogenous Ang-(1–7) and the Mas receptor is still debated in conflicting reports on kidney injury models, treatment with Ang-(1–7) remains a plausible therapeutic strategy in obstructive nephropathy. Further investigations in this field are required to test the effect of Ang-(1–7) treatment on another disease models or various species, as well as the modulatory interaction between classical and nonclassical RAAS in kidney injury.
